# Lipidomic Signature of Plasma and Synovial Fluid in Patients with Osteoarthritis: Putative Biomarkers Determined by UHPLC-QTOF-ESI+MS

**DOI:** 10.3390/diagnostics14161834

**Published:** 2024-08-22

**Authors:** Stefan Iulian Stanciugelu, Jenel Marian Patrascu, Jenel Marian Patrascu, Carmen Socaciu, Andreea Iulia Socaciu, Diana Nitusca, Catalin Marian

**Affiliations:** 1 Doctoral School, Department of Biochemistry and Pharmacology, Victor Babes University of Medicine and Pharmacy, Pta Eftimie Murgu Nr. 2, 300041 Timisoara, Romania; stefan.stanciugelu@umft.ro; 2 Orthopedic and Traumatology Clinic, Timisoara County Emergency Clinical Hospital, B-dul L Rebreanu Nr. 156, 300723 Timisoara, Romania; patrascu.jenel@umft.ro (J.M.P.); jenel.patrascu@umft.ro (J.M.P.J.); 3 Department of Orthopedics and Trauma, Victor Babes University of Medicine and Pharmacy, Pta Eftimie Murgu Nr. 2, 300041 Timisoara, Romania; 4 BIODIATECH, Research Center for Applied Biotechnology in Diagnosis and Molecular Therapy, 400478 Cluj-Napoca, Romania; carmen.socaciu@usamvcluj.ro; 5 Department of Occupational Health, Iuliu Hateganu University of Medicine and Pharmacy, Str. Victor Babes Nr. 8, 400347 Cluj-Napoca, Romania; andreeaiso@gmail.com; 6 Department of Biochemistry and Pharmacology, Victor Babes University of Medicine and Pharmacy, Pta Eftimie Murgu Nr. 2, 300041 Timisoara, Romania; cmarian@umft.ro; 7 Center for Complex Networks Science, Victor Babes University of Medicine and Pharmacy, Pta Eftimie Murgu Nr. 2, 300041 Timisoara, Romania

**Keywords:** lipidomics, metabolomics, osteoarthritis, diagnostic, biomarkers

## Abstract

Background: Osteoarthritis (OA) is a prevalent joint condition causing pain and disability, especially in the elderly. Currently, OA diagnosis relies on clinical data and imaging, but recent interest in metabolomics suggests that early biochemical changes in biofluids, particularly synovial fluid (SF), could enable an earlier diagnosis and understanding of the disease. Methods: In this regard, we conducted a lipidomics study in 33 plasma and SF samples from OA patients and 20 OA-free controls to assess the diagnostic value of various lipid metabolites, using UHPLC-QTOF-ESI+MS. Results: In plasma samples, 25 metabolites had area-under-the-curve (AUC) values higher than 0.9, suggesting a very good diagnostic potential for phosphatidic acid PA (16:0/16:0), PA (34:0), phosphatidylethanolamine PE (34:2), glucosylceramide, phosphatidylcholine PC (32:1), and other metabolites while in SF 20, metabolites had AUC values higher than 0.8, the vast majority belonging to lipid metabolism as well. Conclusions: Although the results align with the previous literature, larger cohort studies are necessary to confirm the diagnostic value of the lipid metabolites.

## 1. Introduction

Osteoarthritis (OA) is one of the most common joint conditions, being the main cause of pain and disability in the elderly. OA is a multifactorial disease involving many risk factors: obesity, gender, trauma, repetitive joint use, genetics, hereditary metabolic diseases, muscle weakness, pre-existing orthopedic disorders, rheumatoid polyarthritis, intra-articular crystal deposits, bone turnover, and blood clotting. The diagnosis of OA is established based on clinical data, which usually develops after the appearance of structural changes [1]. Unfortunately, there are no available tests to confirm an early diagnosis, and the severity of this disease is determined by imaging or arthroscopy [2,3].

Recently, there has been a growing interest in identifying the biochemical signature of OA. Because changes in the metabolic profile occur frequently before alterations in the genome or proteome, metabolomics analysis could allow for the early identification of biochemical changes to establish early diagnosis, disease stage, and therapeutic targets and provide a better understanding of the disease’s pathogenesis [4]. Previous metabolomics studies of OA have frequently used biofluids as a substrate for a minimally invasive approach, such as urine, serum, and synovial fluid (SF). SF is a plasma ultrafiltrate, but it also contains molecules that are produced by joint tissue cells, which serve as media for nutrients and as lubricants for the joint surface. Being located inside the joint cavity, in direct contact with the joint tissues, and with OA being a condition affecting the entire joint, SF is recognized as the most important biofluid in the evaluation of the metabolomic profile of OA [5,6,7,8].

The vast majority of the metabolomic studies of OA are focused on lipid metabolism, as lipidomics could offer additional information related to dysfunctions. To date, few studies have evaluated the metabolic profile of patients with OA using SF as a biological specimen, mostly being focused on blood-derived samples, such as plasma or serum [9].

Mass spectrometry (MS) is the most sensitive detection method, with the widest coverage of the metabolome. It is usually necessary to be coupled with chromatographic separation techniques—gas chromatography (GC) and/or liquid chromatography (LC). LC-MS is usually used for biofluid analysis, with both positive and negative ion detection modes, using standard protocols [10]. The small number of studies that have used LC-MS in the evaluation of SF in OA have conducted global or targeted metabolomics to identify specific biomarkers, differentiate subgroups or phenotypes, and better understand the pathogenesis of the disease. Fewer studies have used NMR in assessing the metabolic profile of SF in patients with OA [11].

Lipids such as sphingolipids, phosphatidic acids, lysophosphatidic acid, and bis-monoacylglycerolphosphates are known to be bioactive molecules, but their presence and function in SF are yet to be understood. Only a few studies have evaluated the lipid profile of SF in OA using MS. Kosinska et al. (2013) reported an increased concentration of phospholipids species in SF of patients with OA compared to controls and differences in this category of metabolites related to OA stage using electrospray ionization tandem mass spectrometry (ESI-MS). These changes could lead to altered joint lubrication, the production of reactive oxygen species (ROS), and scavenging the activity of SF and can modulate the inflammatory state of the joints [12].

Another lipidomics study quantified the composition of sphingolipids and minor glycerophospholipid species in the SF of patients with early and late stages of OA. Several lipid species were significantly increased in different stages of OA, compared to normal SF, and 21 species of lipids were different between early OA and advanced OA. These changes could lead to the development of disease-specific diagnostic and staging biomarkers [13].

Therefore, our study aims to identify specific lipidomic signatures of blood plasma and SF from patients with confirmed OA, using UHPLC-QTOF-ESI+MS technology coupled with statistics, for potential biomarker discovery in terms of OA diagnosis and staging.

## 2. Materials and Methods

### 2.1. Study Subjects and Sample Collection

This study included 53 subjects in total, out of which 33 were patients previously diagnosed with OA (mostly knee OA) and 20 were OA-free controls. Blood and SF were collected from the subjects at the Orthopedic and Traumatology Clinic from Timisoara County Emergency Clinical Hospital, Romania. Every patient had given their informed consent for the use of their biological samples. This study was carried out following the 1964 Declaration of Helsinki and its subsequent amendments, which was authorized by our institution’s Ethics Committee (Ethics Committee of the University of Medicine and Pharmacy “Victor Babes” Timisoara, Romania, approval code no 08/28.02.2020, approval date 28 February 2020). Venous blood was drawn into tubes coated with EDTA. The plasma was separated straightaway by centrifugation (15 min, 2000× *g*) and was kept frozen (−80 °C) for further testing. Orthopedic specialists used routine arthrocentesis methods to collect SF samples. Table 1 displays the clinical and demographic details of the 53 participants in this study.

### 2.2. Sample Processing

A volume of 0.8 mL of solvent mix was added to 0.2 mL plasma (methanol/acetonitrile/methyl tert-butyl ether (1:1:0.25)). The mix was then vortexed for 30 s and kept at −20 °C for 24 h for protein precipitation. After defrosting, the tubes were centrifuged at 12,500× *g* for 10 min. Collection of the supernatant was then performed, which was subsequently filtered through 0.2 µm PTFE filters and introduced in the autosampler vials for further metabolomic analyses. We added 0.75 mL mix of methanol/acetonitrile (1:1) and 0.25 mL of acetone to a volume of 0.25 mL of SF (the supernatant after the centrifugation at 12,500 rpm). The mix was subsequently vortexed for 30 s and kept at −20 °C for 24 h. After defrosting, the samples were centrifuged at 12,500× *g* for 10 min and the supernatant was kept in an ultrasonic bath at 35 °C to eliminate the acetone residue. After filtration through 0.2 µm nylon filters, the samples were introduced in the autosampler vials for analysis.

### 2.3. UHPLC-QTOF-ESI+-MS Analysis

Using a Thermo Scientific HPLC UltiMate 3000 system with a Dionex Ultimate quaternary pump delivery and ESI+-QTOF-MS detection, the UHPLC-MS analysis was carried out on a Bruker Daltonics MaXis Impact device (Bruker GmbH, Bremen, Germany). A C18 reverse-phase column was used (Kinetex, UPLC C18, Phenomenex, Torrance, CA, USA) (5 µm, 4.6 × 150 mm), set at 25 °C and at a flow rate of 0.8 mL/min. There was a 25 μL injection volume. Eluent A (water containing 0.1% formic acid) and eluent B (methanol/acetonitrile/isopropanol, 1:1:1, containing 0.1% formic acid) comprised the gradient that represented the mobile phase. Together, 70% A (min 0), 30% A (min 4), 0% A (min 7), 30% A (min 10), 70% A (min 13), and 70% A for two minutes made up the gradient system. The entire run time was 15 min.

The mass range covered by the MS parameters was 100–1000 Da. The drying gas flow was set at 12 L/min, the temperature of the drying gas was set at 300 °C, and the pressure of the nebulizing gas was set at 2.8 bar. A sodium formate calibration was carried out prior to every chromatographic run. Specific software from Bruker Daltonics—Chromeleon 7.3.2, TofControl 3.2, Hystar 3.2, and Data Analysis 4.2—was utilized for the instrument’s control and data processing. The resolution setting for this untargeted analysis was Full Scan mode since it can capture more metabolic features.

### 2.4. Statistics

Data analysis was used to process the following data: First, the individual Total Ion Chromatograms (TICs) were registered. After that, they were converted to Base Peak Chromatograms (BPCs), and the Find Molecular Features (FMF) tool was used to record the compound spectra. The retention time, peak regions and intensities, signal/noise (S/N) ratio for each component, and *m*/*z* values for each metabolite were all included in the table that was derived from the FMF matrix. Both molecules with S/N values less than 10 and retention times less than 1.6 min (the LC column’s empty volume) were removed. The initial step of the statistical analysis involved saving the matrix as an Excel file, which contained the peak intensities (higher than 10,000) and *m*/*z* [M + H^+^] values for each sample. Using the tools available at https://www.bioinformatics.org/bioinfo-af-cnr/NEAPOLIS/, the *m*/*z* values were aligned (accessed on 8 July 2024). The ability to compute the mean intensity values and standard deviation for every *m*/*z* value was provided by the aligned matrix.

Second, the completed aligned matrix was loaded into the web software Metaboanalyst 6.0 after being converted to a csv file (https://www.metaboanalyst.ca/; accessed on 8 July 2024). For the sample normalization, normalization by sum was chosen. In addition, log transformation and Pareto scaling were chosen for data transformation and scaling, respectively.

For the multivariate statistics, we used the matrices including 245 *m*/*z* values in plasma and 272 values in SF, respectively. The experimental *m*/*z* values were compared with the average of the theoretical *m*/*z* values from the Human Metabolome DataBase (HMDB) and Lipidmaps databases, the accuracy of theoretical–experimental *m*/*z* values being below 30 ppm (±0.01 Da). Then, utilizing the two most pertinent databases, LIPID MAPS^®^ Lipidomics Gateway (CA, USA), the most relevant compounds that may be thought of as possible biomarkers were identified (https://www.lipidmaps.org/databases/lmsd/overview; accessed on 9 July 2024) and Human Metabolome Database (https://hmdb.ca/; accessed on 9 July 2024).

Lastly, the multivariate analysis was represented by partial least squares discriminant analysis (PLSDA) and VIP scores, Fold change values, Volcano plots, and Random Forest (Mean Decrease Accuracy) for prediction, finding correlations between samples and between variables (*m*/*z* values). Using biomarker analysis, the Receiver Operating Curves (ROCs) were obtained and the values of areas under ROC curves (AUCs) were ranked according to their sensitivity/specificity. The Enrichment Analysis was applied based on the identified cohorts of molecules.

## 3. Results

The clinical and demographic information for both the OA-affected patients and the OA-free controls is shown in Table 1. The mean age discrepancy between the cases and controls (64.5 years vs. 35.2, respectively) could not be adjusted due to the small sample size. Both groups contained both male and female members, and the majority of them (66.7% of the OA patients and 70% of the healthy controls) were urban residents. Nearly eighty-five percent of the study’s participants had been diagnosed with knee OA, while the controls did not have OA and were primarily admitted to the clinic because of bone fractures or ligament ruptures caused by accidents.

**Figure 1:**
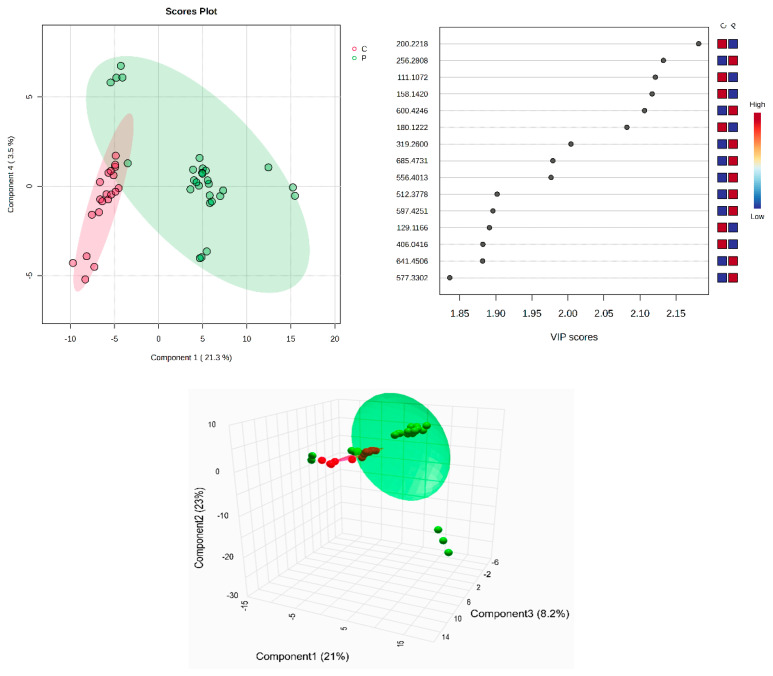
(**Left**) partial least squares discriminant analysis (PLSDA) plot with sample identification, showing the discrimination between C and P groups; (**right**) PLSDA loadings plot, showing the VIP scores of the main 15 molecules selected as representative for the discrimination between groups C and P; (**bottom**) scatter 3D considering the first 3 components released from Metaboanalyst 6.0.

All identified metabolites, either the common ones found in plasma and SF or the molecules identified only in plasma or in SF, can be seen in Supplementary Table S1A–C. The tables include the *m*/*z* values for the precursor ion (adduct [M + H^+^], identified as putative biomarkers by comparison with the average isotopic mass and a mass tolerance of 0.05 Da, according to the HMDB and LipidMaps databases (IDs included). Their identification, with putative names, has been carried out using the HMDB and LipidMaps databases. The multivariate analysis of the plasma samples assessed by supervised PLSDA evaluated the co-variance for the first five components. The explained co-variance of groups C (controls) and P (patients) was 24.8%, the discrimination being significant (Figure 1, left). From the PLSDA loadings plot, we selected the most relevant molecules that could be considered responsible for the discrimination. Figure 1 (right) represents the *m*/*z* values of these molecules and the VIP scores above 1 for the top 15 molecules, as a measure of their significance. The red and blue colors reflect the relative variation of their values in the C versus P group.

According to the PLSDA plot, the P group was less homogeneous than the C group. The cross-validation algorithm (when the first four components were considered) indicated an accuracy of over 0.9, a maximal value of R2 = 0.95, and Q2 values over 0.7, suggesting a good predictability for this model.

**Figure 2:**
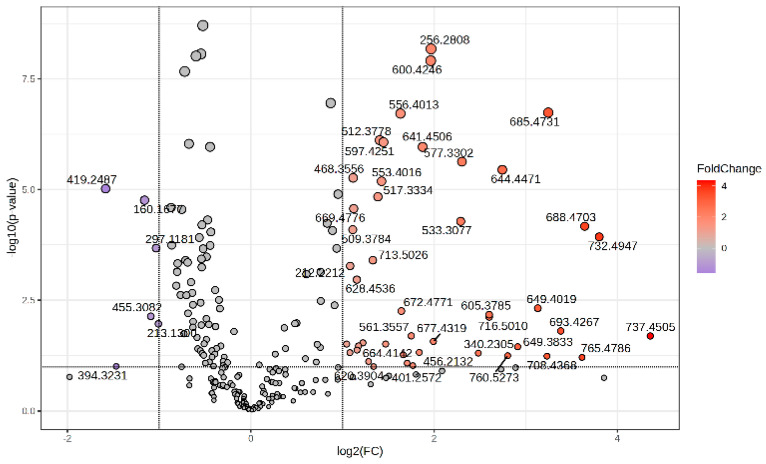
Volcano plot representing the *m*/*z* values of molecules with increased MS intensity levels in the P group compared to the C group.

The Volcano plot (Figure 2) represents the *m*/*z* values of molecules with higher peak intensity in the P group (log_2_ FC > 0) and decreased levels (log_2_ FC < 0) compared to controls (group C).

The Metaboanalyst 6.0 program states that the receiver operating characteristic (ROC) curve is a helpful tool for assessing the accuracy of the diagnosis in biomarker analysis. The area under the ROC curve (AUC) is a key component of several biomarker combination techniques. This characteristic made it possible to evaluate each molecule’s sensitivity against specificity and identify each one as a potential biomarker. Higher AUC values around 1 indicate a higher likelihood of a particular molecule being regarded as a biomarker. Table 2 displays the probable identification, *m*/*z* value, AUC value, *p*-values, and log_2_FC values for every identified molecule, along with its variance across the P and C groups.

Significantly high AUC values above 0.9 were obtained for 25 molecules that might be considered potential biomarkers for OA diagnosis; these molecules belong to various lipid classes. These data confirm that lipid molecules, mainly long-chain glycerophosphates (PA) and glycerophospholipids (PC and PE) can be considered predictive molecules with good diagnostic values for OA detection. Glucosamine is also a particularly good biomarker related to OA, and in our study, its values in the P group were significantly lower than in the C group.

**Figure 3:**
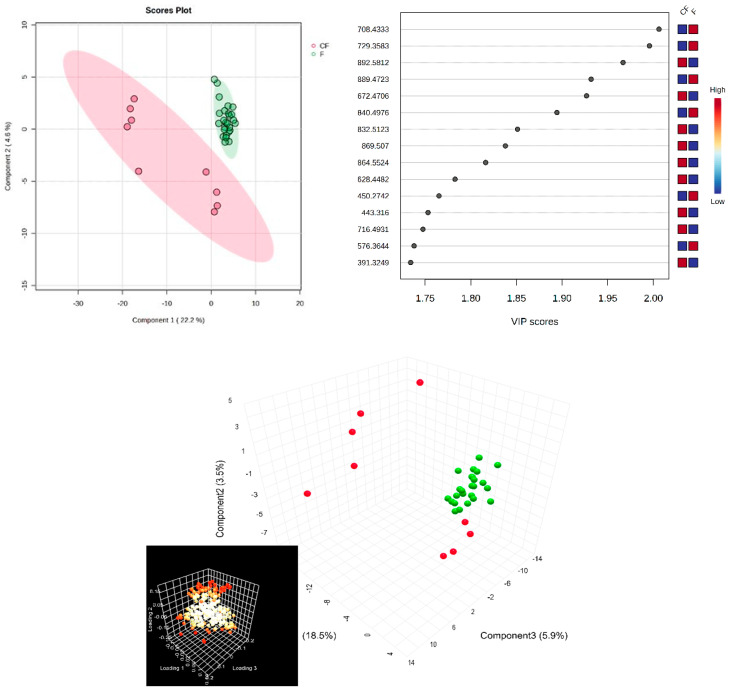
(**Left**) partial least squares discriminant analysis (PLSDA) plot with sample identification, showing the discrimination between groups; (**right**) PLSDA loadings plot, showing the VIP scores of the main 15 molecules selected as representative for the discrimination between groups; (**bottom**) scatter 3D for SF (considering first 3 components); in the bottom left corner, there is the loadings representation, released from Metaboanalyst 6.0.

Next, in the multivariate analysis of the SF samples, via supervised PLSDA, the co-variance for the first five components was similarly evaluated. The explained co-variance of groups was 22%, the discrimination being significant (Figure 3, left). The right of Figure 3 represents the loadings graph showing the top 15 molecules (*m*/*z* values) and their corresponding VIP scores above 1 as a measure of discrimination between groups.

According to the PLSDA plot, in this case, the control group was less homogeneous than the patient group, and this can be explained by the metabolic diversity of the control group.

Next, a biomarker analysis was also performed, using MetaboAnalyst 6.0. Table 3 shows the *m*/*z* values and putative identifications, AUC values, *p*-values, and log_2_FC values for each molecule identified, as well as the variation in the comparison groups.

As in the case of blood plasma, many glycerophospholipids derivatives (PS, PI, PE, and PG), lyso-derivatives (LPC), and free fatty acids (myristic acid) were identified with AUC values > 0.8. Prostaglandin derivatives can be considered important biomarkers due to their involvement in inflammation.

**Figure 4:**
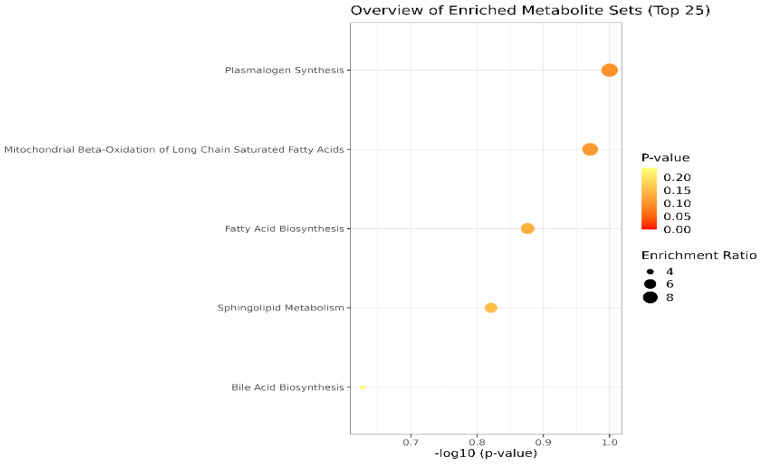
Main metabolic pathways affected in OA, according to the cohort of molecules identified in blood plasma and SF (*p*-values < 0.1). The dimension and color of the circles’ graphic illustrate the impact of the first 5 metabolic pathways.

Lastly, the cohort of molecules separated and identified in plasma and SF samples were subjected to the Pathway Enrichment Analysis, provided by Metaboanalyst 6.0. Figure 4 shows an overview of the metabolite sets (top 25) found in plasma and SF. The most relevant are the pathways related to plasmalogen synthesis, molecules with an ester (acyl group) linked lipid at the sn-2 position of the glycerol backbone, chemically designated as 1-0(1Z-alkenyl)-2-acyl-glycerophospholipids. Moreover, mitochondrial beta-oxidation, fatty acid, and sphingolipid metabolism are affected in OA, according to the inputs of our study.

## 4. Discussion

In this study, we investigated the lipidomic signature of OA in 53 subjects (33 OA patients and 20 healthy controls) to identify potential minimally invasive diagnostic biomarkers for the detection of this disease. As it is well known that the lipid metabolism suffers alterations in the early phases of the OA pathogenesis, we identified lipid derivatives with very good diagnostic accuracies (AUC > 0.9), with significantly altered levels in plasma and SF of OA patients relative to controls.

While there are not many human studies examining the lipidomic profile in relation to OA development in SF, our findings generally agree with other publications that have been published. Gas chromatography/time-of-flight mass spectrometry (GC/TOF-MS) has been used by several authors to examine the metabolic profile of SF in patients with early (KL grade 1 or 2) vs. late (KL grade 3 or 4) knee OA. Subsequently, hierarchical clustering and orthogonal partial least squares discriminant analyses were performed. Patients with late-stage knee OA were found to have elevated levels of several lipids, such as arachidonic acid, palmitoleic acid, linoleic acid, oleic acid, palmitic acid, and stearic acid, in addition to other metabolites [14,15,16].

Furthermore, it has been shown that SF depends on lipid species to lubricate joints and lower friction; as a result, this dysregulation probably represents the inflammatory and lubricating states of the joint tissue after injury. Furthermore, the dysregulation of glycolysis, the tricarboxylic acid cycle, fatty acid production, oxidation, and degradation in injured participants may represent the health and functioning of mitochondria in different joint tissues after injury. Energy is produced by both glucose and fatty acids in functioning mitochondria but in malfunctioning mitochondria, the preference is for fatty acids over glucose. Fatty acid buildup can result in decreased adenosine triphosphate (ATP) synthesis, a compromised stress response, an increase in reactive oxygen species, apoptosis, and other factors that when combined can cause systemic irreversible damage [17,18,19].

In addition, in support of these findings, Wu et al. (2017) investigated possible alterations in the lipid profile of mice SF, finding significant dysregulations, suggesting that the investigation of the lipid metabolism in OA is crucial for the early detection of the disease. The authors found that there was a positive correlation between the levels of adiponectin and the serum levels of ω-3 PUFAs but a negative correlation with OA and wound size. Conversely, the majority of ω-6 PUFAs showed favorable associations with inflammatory adipokines, OA, and poor healing. It is noteworthy that there was an inverse correlation between joint degradation and the amounts of palmitoleic acid and pentadecylic acid (C15:0, an odd-chain SFA). Because injuries may result in mitochondrial malfunction, which allows fatty acids to enter the mitochondria and become the main energy source, it has been shown that lipid metabolism plays a significant role in joint health [20].

Other plasma metabolomics studies in OA patients identified upregulated molecules such as palmitoleic acid (C16:1), carnitine C20:2, hypoxanthine, xanthosine, and N-α-acetyl-L-asparagine, which were positively correlated with knee OA [21]. In addition, in the patellar, lateral, and medial compartments, the reduction in cartilage volume was 1.98 ± 0.46%, 1.06 ± 0.58%, and 1.34 ± 0.72% annually, respectively. According to these results, there was a 0.12 ± 0.02% annual decrease in patellar cartilage volume linked with the increased ratios of hexadecenoylcarnitine (C16:1) to tetradecanoylcarnitine (C14) and C16:1 to dodecanoylcarnitine (C12) (*p* < 3.03 × 10^−6^). These findings indicated that the loss of patellar cartilage was related to changes in long-chain fatty acid β-oxidation. The ratios of C16:1 to C14 and C12 may be utilized to predict long-term cartilage deterioration, although further research is required [21,22].

It is interesting to note that patients with metabolic problems may have aberrant plasma concentrations of carnitine and acylcarnitine due to anomalies in the metabolic enzymes or transport proteins involved in fatty acid and amino acid metabolism. It is well established that the main role of carnitine is to combine with fatty acids or deamination products of branched-chain amino acids to form acylcarnitine. After leaving the cytoplasm, these metabolites are transferred to the mitochondria, where beta-oxidation completely breaks them down and releases energy. Thus, abnormalities in one or more acylcarnitine levels are indicative of diseases related to the metabolism of fatty acids or branched-chain amino acids [23].

Nevertheless, our research was limited in terms of population size (53 subjects) and had an age mismatch between patients and controls that could not be adjusted, which represents the main limitations of the current work. Although the results are consistent with previous literature reports, for the lipid metabolites to have a more reliable diagnostic value, bigger cohort studies are required to substantiate our findings, and further targeted lipidomics studies are warranted.

## 5. Conclusions

We have shown that the lipid profile of OA patients is highly altered and that the affected metabolites could represent novel biomarkers for the detection of the disease. We have found 25 molecules with very high diagnostic accuracies (AUC > 0.9) that were significantly dysregulated in plasma samples of OA patients compared to OA-free controls and 20 molecules with high AUC values (>0.8) in SF. However, given the small-scale design of our study, future, large-scale targeted lipidomics studies are required to confirm and further validate our findings.

## Figures and Tables

**Table 1:** Demographic and clinical characteristics of OA patients and controls.

Characteristics	Patients (*n* = 33)	Controls (*n* = 20)
Age (±SD)	64.5 (±8.0)	35.2 (±16.4)
Biological sex, *n* (%)		
Female	16 (48.5%)	10 (50.0%)
Male	17 (51.5%)	10 (50.0%)
Geographic area, *n* (%)		
Urban	22 (66.7%)	14 (70.0%)
Rural	11 (33.3%)	6 (30.0%)
Diagnosis, *n* (%)		
Knee OA	28 (84.8%)	
Hip joint OA	3 (9.1%)	
Shoulder OA	2 (6.1%)	

**Table 2:** The *m*/*z* values, area-under-the-curve (AUC) values, *p*-values, and log_2_FC values for the putative biomarkers in blood plasma of OA patients compared to controls.

*m*/*z* Value	Identification	AUC	*p*-Value	Log_2_FC	Tendency
649.4019	PA (16:0/16:0)	0.977	5.141 × 10^−3^	−2.974	P > C
677.4319	PA (34:0)	0.973	2.772 × 10^−2^	−1.767	P > C
693.4267	PA (36:6)	0.957	1.623 × 10^−2^	−3.242	P > C
605.3785	PA (26:4;O2)	0.954	7.273 × 10^−3^	−2.457	P > C
180.1222	Glucosamine	0.952	1.544 × 10^−8^	0.378	P < C
158.1420	Tiglylglycine	0.943	6.066 × 10^−9^	0.290	P < C
716.5010	PE (34:2)	0.941	8.020 × 10^−3^	−2.431	P > C
688.4703	PE (32:2)	0.939	8.144 × 10^−5^	−3.647	P > C
111.1072	Hydroquinone	0.934	4.864 × 10^−9^	0.240	P < C
200.2218	Octenoylglycine	0.929	9.512 × 10^−10^	0.257	P < C
737.4505	PA (39:5)	0.929	2.121 × 10^−2^	−4.185	P > C
644.4471	Glucosylceramide (d18:1/12:0)	0.923	4.578 × 10^−6^	−2.840	P > C
664.4142	PE (30:0)	0.921	8.399 × 10^−2^	−1.351	P > C
732.4947	PC (32:1)	0.921	1.132 × 10^−4^	−4.078	P > C
765.4786	PA (41:5)	0.920	6.297 × 10^−2^	−3.328	P > C
172.1554	L-Homocysteine sulfate	0.909	2.154 × 10^−5^	0.447	P < C
235.1505	5-Methoxytryptophan	0.904	8.310 × 10^−5^	0.163	P < C
633.4083	PA (O-16:0/16:1)	0.904	3.011 × 10^−2^	−1.246	P > C
149.0811	L-2-Hydroxyglutaric acid	0.902	6.590 × 10^−1^	0.057	P < C
406.0416	12-HETE-GABA	0.902	6.884 × 10^−7^	0.165	P < C

Abbreviations: PA = phosphatidic acid; PE = phosphatidylethanolamine; PC = phosphatidylcholine.

**Table 3:** The *m*/*z* values, AUC values, *p*-values, and log_2_FC values for the putative biomarkers in SF of OA patients relative to controls.

*m*/*z* Value	Identification	AUC	*p*-Value	Log_2_FC	Tendency
427.2684	N-stearoylarginine	0.919	5.116 × 10^−4^	0.710	P < C
123.0358	Diammonium Oxalate	0.897	5.153 × 10^−3^	0.540	P < C
892.5812	PS (44:6)	0.897	5.305 × 10^−6^	1.656	P < C
229.1307	Myristic acid	0.893	3.945 × 10^−4^	0.571	P < C
889.4723	PG (44:1)	0.880	8.980 × 10^−6^	−1.106	P > C
672.4706	PE (31:3)	0.863	9.671 × 10^−6^	0.595	P < C
576.3644	LPC (22:2)	0.859	1.124 × 10^−4^	−0.946	P > C
708.4333	Cer (d18:0/28:0)	0.859	2.846 × 10^−6^	−1.189	P > C
801.4294	PI (32:5)	0.855	2.528 × 10^−4^	−0.991	P > C
845.4516	PI (35:4)	0.855	2.049 × 10^−4^	−0.918	P > C
620.3867	PE (P-16:0/12:0)	0.842	8.591 × 10^−4^	−1.069	P > C
713.3851	PI (25:0)	0.842	3.234 × 10^−4^	−1.036	P > C
729.3583	PI 23:2;O3	0.838	3.370 × 10^−6^	−1.331	P > C
664.4095	PE (30:0)	0.833	2.137 × 10^−3^	−1.060	P > C
116.0625	L-Proline	0.829	3.572 × 10^−3^	0.722	P < C
522.5724	LysoPC (18:1)	0.825	2.013 × 10^−4^	−0.942	P > C
897.5344	PI (39:6)	0.825	1.581 × 10^−3^	0.613	P < C
368.4051	N-oleoyl GABA	0.821	5.418 × 10^−3^	−0.643	P > C
840.4976	PE (44:10)	0.821	1.537 × 10^−5^	−1.097	P > C
285.2153	Stearic acid	0.816	1.392 × 10^−2^	0.689	P < C

Abbreviations: PS = phosphatidylserine; PG = phosphatidylglycerol; PE = phosphatidylethanolamine; LPC = lysophosphatidylcholine; PI = phosphatidylinositol; Cer = ceramide; GABA = gamma-aminobutyric acid.

## Data Availability

All data are available in the manuscript.

## Supplementary Materials

The following supporting information can be downloaded at: https://www.mdpi.com/article/10.3390/diagnostics14161834/s1, Table S1A. Metabolites found in both plasma and synovial fluid, with *m*/*z* values for the precursor ion (adduct [M + H^+^], identified as putative biomarkers by comparison with the average isotopic mass and a mass tolerance of 0.05 Da, according to HMDB and LipidMaps databases (IDs included); Table S1B. Metabolites found in plasma with *m*/*z* values for the precursor ion (adduct [M + H^+^], identified as putative biomarkers by comparison with the average isotopic mass and a mass tolerance of 0.05 Da, according to HMDB and LipidMaps databases (IDs included). Plasma putative biomarkers of differentiation between control groups C and P, selected according to three statistic algorithms: PLSDA (VIP values above 1.5), Random Forest (RF) with MDA values above 0.0005, and AUC values above 0.900; Table S1C. Metabolites found in the synovial fluid with *m*/*z* values for the precursor ion (adduct [M + H^+^], identified as putative biomarkers by comparison with the average isotopic mass and a mass tolerance of 0.05 Da, according to HMDB and LipidMaps databases (IDs included)). Putative biomarkers of differentiation between controls and patients in synovial fluid were selected according to three statistic algorithms: PLSDA (VIP values above 1.7), Random Forest (RF) with MDA values above 0.0002, and AUC values above 0.800.

## References

[1] Jiang Y. (2022). Osteoarthritis year in review 2021: Biology. Osteoarthr. Cartil..

[2] Kohn M.D., Sassoon A.A., Fernando N.D. (2016). Classifications in Brief: Kellgren-Lawrence Classification of Osteoarthritis. Clin. Orthop. Relat. Res..

[3] Slattery C., Kweon C.Y. (2018). Classifications in Brief: Outerbridge Classification of Chondral Lesions. Clin. Orthop. Relat. Res..

[4] Li J.T., Zeng N., Yan Z.P., Liao T., Ni G.X. (2021). A review of applications of metabolomics in osteoarthritis. Clin. Rheumatol..

[5] Abdelrazig S., Ortori C.A., Doherty M., Valdes A.M., Chapman V., Barrett D.A. (2021). Metabolic signatures of osteoarthritis in urine using liquid chromatography-high resolution tandem mass spectrometry. Metabolomics.

[6] Xiao Z., Zhang Z., Huang S., Lon J.R., Xie S. (2022). Metabolic Profiling of Serum for Osteoarthritis Biomarkers. Dis. Markers.

[7] Zheng K., Shen N., Chen H., Ni S., Zhang T., Hu M., Wang J., Sun L., Yang X. (2017). Global and targeted metabolomics of synovial fluid discovers special osteoarthritis metabolites. J. Orthop. Res..

[8] Nieminen P., Hämäläinen W., Savinainen J., Lehtonen M., Lehtiniemi S., Rinta-Paavola J., Lehenkari P., Kääriäinen T., Joukainen A., Kröger H. (2022). Metabolomics of Synovial Fluid and Infrapatellar Fat Pad in Patients with Osteoarthritis or Rheumatoid Arthritis. Inflammation.

[9] Jónasdóttir H.S., Brouwers H., Kwekkeboom J.C., van der Linden H.M.J., Huizinga T., Kloppenburg M., Toes R.E.M., Giera M., Ioan-Facsinay A. (2017). Targeted lipidomics reveals activation of resolution pathways in knee osteoarthritis in humans. Osteoarthr. Cartil..

[10] Yu S., Zou Y., Ma X., Wang D., Luo W., Tang Y., Mu D., Zhang R., Cheng X., Qiu L. (2024). Evolution of LC-MS/MS in clinical laboratories. Clin. Chim. Acta..

[11] Zhang D., Zhang Y., Xia S., Shen P., Yang C. (2024). Metabolic profiling of synovial fluid in human temporomandibular joint osteoarthritis. Front. Immunol..

[12] Kosinska M.K., Liebisch G., Lochnit G., Wilhelm J., Klein H., Kaesser U., Lasczkowski G., Rickert M., Schmitz G., Steinmeyer J. (2013). A lipidomic study of phospholipid classes and species in human synovial fluid. Arthritis Rheum..

[13] Kosinska M.K., Liebisch G., Lochnit G., Wilhelm J., Klein H., Kaesser U., Lasczkowski G., Rickert M., Schmitz G., Steinmeyer J. (2014). Sphingolipids in human synovial fluid—A lipidomic study. PLoS ONE.

[14] Li H., Cui Y., Wang J., Zhang W., Chen Y., Zhao J. (2024). Identification and validation of biomarkers related to lipid metabolism in osteoarthritis based on machine learning algorithms. Lipids Health Dis..

[15] Loef M., van de Stadt L., Böhringer S., Bay-Jensen A.C., Mobasheri A., Larkin J., Lafeber F.P.J.G., Blanco F.J., Haugen I.K., Berenbaum F. (2022). The association of the lipid profile with knee and hand osteoarthritis severity: The IMI-APPROACH cohort. Osteoarthr. Cartil..

[16] Kim S., Hwang J., Kim J., Ahn J.K., Cha H.S., Kim K.H. (2017). Metabolite profiles of synovial fluid change with the radiographic severity of knee osteoarthritis. Jt. Bone Spine.

[17] Welhaven H.D., Welfley A.H., Brahmachary P., Bergstrom A.R., Houske E., Glimm M., Bothner B., Hahn A.K., June R.K. (2024). Metabolomic Profiles and Pathways in Osteoarthritic Human Cartilage: A Comparative Analysis with Healthy Cartilage. Metabolites.

[18] Blanco F.J., Lopez-Armada M.J., Maneiro E. (2004). Mitochondrial dysfunction in osteoarthritis. Mitochondrion.

[19] Blanco F.J., Rego I., Ruiz-Romero C. (2011). The role of mitochondria in osteoarthritis. Nat. Rev. Rheumatol..

[20] Wu C.L., Kimmerling K.A., Little D., Guilak F. (2017). Serum and synovial fluid lipidomic profiles predict obesity-associated osteoarthritis, synovitis, and wound repair. Sci Rep..

[21] Wang X., Cai W., Liu Y., Lu Y., Liu M., Cao X., Guo D. (2023). Exploring biomarkers associated with severity of knee osteoarthritis in Southern China using widely targeted metabolomics. BMC Musculoskelet. Disord..

[22] Xie Z., Aitken D., Liu M., Lei G., Jones G., Cicuttini F., Zhai G. (2022). Serum Metabolomic Signatures for Knee Cartilage Volume Loss over 10 Years in Community-Dwelling Older Adults. Life.

[23] Liao Z., Han X., Wang Y., Shi J., Zhang Y., Zhao H., Zhang L., Jiang M., Liu M. (2023). Differential Metabolites in Osteoarthritis: A Systematic Review and Meta-Analysis. Nutrients.

