# Supplementary material for: Lipidomic Signature of Plasma and Synovial Fluid in Patients with Osteoarthritis: Putative Biomarkers Determined by UHPLC-QTOF-ESI+MS
Source: Diagnostics (Basel). 2024 Aug 22;14(16):1834. doi: 10.3390/diagnostics14161834 (PMC11354166; doi:10.3390/diagnostics14161834)
Supplement: Supplementary file 1 [file diagnostics-14-01834-s001.zip › diagnostics-3158075-supplementary.pdf]

Table S1A. Metabolites found in both plasma and synovial fluid, with m/z values for the precursor ion (adduct [M+H+], identified as putative biomarkers by comparison with the average isotopic mass and a mass tolerance of 0.05 Da, according to HMDB and LipidMaps databases (IDs included).

m/z (M+H⁺)	Identification	ID (HMDB/LMDB)
125.9759	Taurine	HMDB0000251
143.9874	Proline betaine	HMDB0004827
149.0532	2-hydroxyglutaric acid	HMDB0000606
158.1420	Tiglylglycine	HMDB0000959
172.1554	L-Homocysteine sulfate	HMDB0002238
173.1421	Capric acid C10:0	HMDB0000511
200.2239	O-Phosphothreonine	HMDB0251566
202.2044	Cysteine-S-sulfate	HMDB0000731
203.0210	Spermine	HMDB0001256
203.0388	Sebacic acid	HMDB0000792
205.0778	Trytptophan	HMDB0000929
205.1057	2-hydroxy-7-methyl-Octanedioic acid	LMFA01170136
212.2212	Creatine phosphate	HMDB0001511
213.1350	Traumatin C12:2;O	LMFA01060167
214.2368	Indoxyl sulfate	HMDB0000682
216.2156	Propenoylcarnitine	HMDB0013124
217.1409	3-Hydroxydodecanoic acid	LMFA01050363
228.2514	Deoxycytidine	HMDB0000014
229.1224	Myristic acid	LMFA01010014
230.2308	Butenylcarnitine	HMDB0013126
235.1505	5-Methoxytryptophan	HMDB0002339
239.1537	Pentadecynoic acid (C15:2)	LMFA01030581
242.2685	Tetrahydrobiopterin	HMDB0000027
243.1170	Thymidine	HMDB0000273
245.0616	Uridine	HMDB0000296
249.1866	Hexadecatetraenoic acid C16:4	LMFA01030280
256.2808	Palmitamide	LMFA08010009
258.2599	Glycerophosphocholine	HMDB0000086
271.1695	Estrone	LMST02010004
275.1797	Alfa-androstenol	LMST02020008
277.1961	Stearidonic acid C18:4	LMFA01030357
279.2096	alpha-Linolenic acid C18:3	LMFA01030152
286.2893	Octenoylcarnitine CAR 8:1	LMFA07070014
287.1400	Dehydrotestosteron	LMST02020018
290.2626	O-adipoylcarnitine	LMFA07070086
295.2089	HOTE - C 18:3;O	LMFA02000029

297.2202	9-HODE	HMDB0062652
298.3260	Sphingosine 18:2; O2	LMSP01080010
301.1185	Eicosatetraynoic acid C20:6	LMFA01030692
304.2776	3-hydroxydecanoyl carnitine	HMDB0061636
311.2305	Eicosenoic acid	LMFA01030082
312.3405	(2E,4Z)-decadienoylcarnitine	HMDB0240751
313.2261	Arachidic acid C20:0	LMFA01010020
317.1846	Pregnenolone	LMST02030088
319.2600	Hydroxy-Eicosatetraenoic acid C20:4;O	LMFA01030719
326.9739	N-Oleoylethanolamine	LMFA08040015
332.3074	Tetradecanoylcarnitine	HMDB0005066
335.2033	PGA2	HMDB0001138
337.2110	PGA1	HMDB0002656
340.2305	Dodecadienoylcarnitine	LMFA07070124
340.3693	Oleoyl glycine	LMFA08020082
341.2424	Behenic acid C 22:0	LMFA01010022
342.3482	Dodecenoylcarnitine CAR 12:1	LMFA07070115
347.2906	Corticosterone	LMST02030186
351.2250	Prostaglandin E3	HMDB0002664
353.2359	PGE2	HMDB0060041
354.3849	C16 Sphinganine 1-P	LMSP01050006
355.2412	PGF2a	LMFA03010025
355.3462	10-oxo-docosanoic acid C22:1;O	LMFA01060139
357.2326	PGF1a	HMDB0002685
359.2083	Tetracosapentaenoic acid	LMFA01030820
360.3368	2-Hydroxylauroylcarnitine CAR 12:0;O	HMDB0013164
368.3992	Tetradecadienoylcarnitine CAR 14:2	LMFA07070020
369.3085	Lignoceric acid C 24:0	LMFA01010024
380.3098	C18-Sphingosine 1-phosphate	HMDB0000277
381.2697	Dimethyl PGE2	HMDB0244730
391.3127	12-Ketodeoxycholic acid	HMDB0000328
393.2631	3-Oxocholic acid	HMDB0000502
395.2219	LysoPA(P-16:0)	HMDB0011154
396.4285	Hexadecadienoylcarnitine C16:2	HMDB0240757
399.2833	N-Palmitoyltryptamine	HMDB0040815
401.2572	5,6-trans-25-Hydroxyvitamin D3	HMDB0006721
402.3805	O-(13-carboxytridecanoyl)carnitine CAR 14:1;O2	LMFA07070084
405.2306	Cortisol 21-Acetate	LMST02030093
406.3019	15-HETE-GABA	LMFA08020148
408.3388	N-linolenoyl glutamic acid	LMFA08020214
413.2619	25-Hydroxyvitamin D2	LMST03010030
424.3328	O-linoleoylcarnitine	LMFA07070092

425.1916	Alpha-Tocotrienol	LMPR02020054
427.2559	N-stearoyl arginine	LMFA08020136
437.2506	LPA(18:1)	LMGP10050008
443.3018	Cortisol 21- sulfate	LMST05020020
453.3091	Palmityl myristate	LMFA07010002
457.3169	Sulfolithocholic acid	LMST05020015
463.2646	Arahidonyl Serotonin	LMFA08020141
470.2321	LPS(14:0)	LMGP03050009
487.3244	LPA (22:4)	LMGP10050020
489.2831	Cytidine 5'-Diphosphocholine	HMDB0260340
494.5313	LPC(16:1)	LMGP01050021
496.3842	LPC(16:0)	LMGP01050018
511.3598	LPG(18:1)	LMGP04050006
522.5613	LPC(18:1)	LMGP01050029
533.3077	Linoleyl stearate	LMFA07010152
537.2970	Stearyl stearate	LMFA07010054
540.4077	Cer(d18:0/16:0)	LMSP02020001
576.3673	LysoPC(22:2)	LMGP01050135
581.3221	DG(33:1)	LMGL02010013
584.4308	Cer(d18:0/18:0(2OH))	LMSP02020030
589.3851	DG(34:4)	LMGL02010028
595.3342	DG(34:1)	LMGL02010004
605.3785	PA(26:4;O3)	LMGP20070030
609.3153	DG(35:1)	LMGL02010029
610.5343	Cer(t18:1(6OH)/20:0)	LMSP02010144
620.3904	PC 23:2; O	LMGP20010028
625.3453	Cer(d18:0/22:0)	LMSP02030017
628.4536	Cer(t18:0/20:0(2OH))	LMSP02030017
633.4083	CerPE(d16:1/16:0)	LMSP03020019
638.5632	Cer(t18:1(6OH)/22:0)	LMSP02010142
639.3513	PA(32:5)	LMGP10010061
641.3313	DG(38:6)	LMGL02010130
644.4471	Glucosylceramide (d18:1/12:0)	LMSP0501AA01
649.3833	PA(16:0/16:0)	LMGP10010013
653.3395	18:0 Cholesterol ester	LMST01020007
664.4142	Cer(d18:1/25:0)	LMSP02010013
666.5912	Cer(d18:0/25:0)	LMSP02020051
669.3704	PA(34:4)	LMGP10010065
672.4771	PE(31:3)	LMGP02010392
677.4319	SM(d18:0/14:0)	LMSP03010030
683.3764	PI (22:2;O)	LMGP20050019
685.3851	PA(P-18:0/18:2)	LMGP10030040

688.4703	PE(32:2)	LMGP02010108
693.4267	PA(36:6)	LMGP10010378
708.4368	Cer(d18:0/28:0)	LMSP02020049
713.3923	CerPE(d14:2/24:1)	LMSP03020037
716.5010	PC(P-16:0/16:1))	LMGP01030026
721.4561	PA(38:6)	LMGP10010038
732.4947	PC(16:0/16:1)	LMGP01010566
737.4505	DG (44:0)	LMGL02010258
757.4166	CerPE(d16:2/24:1(2OH))	LMSP03020094
760.5273	PC(34:1)	LMSP02010178
765.4786	PS(18:0/16:0)	LMGP03010888

Table S1B. Metabolites found in plasma with m/z values for the precursor ion (adduct [M+H+], identified as putative biomarkers by comparison with the average isotopic mass and a mass tolerance of 0.05 Da, according to HMDB and LipidMaps databases (IDs included). Plasma putative biomarkers of dif-ferentiation between control group C and P, selected according to three statistic algorithms: PLSDA (VIP values above 1.5), Random Forest (RF) with MDA values above 0.0005 and AUC values above 0.900.

m/z	Statistical evaluation	Identification	Identification ID/Database
111.1072	VIP & AUC	Hydroquinone	HMDB0002434
129.1166	VIP & RF	Pyroglutamine	HMDB0062558
149.0811	AUC	L-2-Hydroxyglutaric acid	HMDB0000694
158.1420	VIP & RF & AUC	Tiglylglycine	HMDB0000959
172.1554	AUC	L-Homocysteine sulfate	HMDB0002238
180.1222	VIP & RF & AUC	Glucosamine	HMDB0001514
200.2218	VIP & RF & AUC	Octenoylglycine	HMDB0094805
235.1505	AUC	5-Methoxytryptophan	HMDB0002339
256.2808	VIP	Palmitamide	HMDB0012273
319.2600	VIP	15-HEPE	HMDB0012273
406.0416	VIP & AUC	12-HETE-GABA	LMFA08020299
509.3784	RF	PG(18:2(9Z,12Z)/0:0)	LMGP04050014
512.3778	VIP	PS(17:0)	LMGP03050030
556.4013	VIP	PS(2-OMe-19:0/0:0)	LMGP03060018
577.3302	VIP	PA(P-16:0/12:0)	LMGP10030001
597.4251	VIP	DG(34:0)	LMST01070001
600.4246	VIP	Cer(t18:0/18:0(2OH))	LMSP02030016
605.3785	RF & AUC	PA (26:4;O2)	LMGP20070029
633.4083	AUC	PA(O-16:0/16:1(9Z))	LMGP10020008
641.4506	VIP & RF	DG(38:6)	LMGL02010132

644.4471	AUC	Glucosylceramide (d18:1/12:0)	HMDB0004969
649.4019	RF & AUC	PA(16:0/16:0)	LMGP10010027
664.4142	AUC	PE(30:0)	LMGP02010301
669.4776	RF	PA (34:4)	LMGP10010590
677.4319	RF & AUC	PA(34:0)	LMGP10010888
685.4731	VIP	PA(35:3)	LMGP10010346
688.4703	RF & AUC	PE (32:2)	LMGP02010108
693.4267	RF & AUC	PA (36:6)	LMGP10010378
716.5010	AUC	PE(34:2)	LMGP02011198
732.4947	AUC	PC(32:1)	LMGP01011479
737.4505	RF & AUC	PA (39:5)	LMGP10010274
765.4786	RF & AUC	PA (41:5)	LMGP10010804

Table S1C. Metabolites found in the synovial fluid with m/z values for the precursor ion (adduct [M+H+], identified as putative biomarkers by comparison with the average isotopic mass and a mass tol-erance of 0.05 Da, according to HMDB and LipidMaps databases (IDs included) Putative biomarkers of differentiation between controls and patients in synovial fluid were selected according to three statistic algorithms: PLSDA (VIP values above 1.7), Random Forest (RF) with MDA values above 0.0002 and AUC values above 0.800.

m/z	Statistical evaluation	Identification	Identification ID/Database
116.0625	AUC	L-Proline	HMDB0251528
123.0358	RF & AUC	Diammonium Oxalate	HMDB0303018
212.2253	RF	Creatine phosphate	HMDB0001511
229.1307	AUC	Myristic acid	LMFA01010014
285.2153	AUC	Stearic acid	HMDB0000827
340.3736	RF	Oleoyl glycine	HMDB0013631
360.3411	RF	3-hydroxydodecanoyl carnitine	HMDB0061638
368.4051	AUC	N-oleoyl GABA	LMFA08020104
380.3177	RF	C18-Sphingosine 1-phosphate	LMSP01050001
391.3249	VIP & RF	12-Ketolithocholic acid	LMST04010155
399.2918	RF	15-hydroxy-pentacosanoic acid	LMFA01050217
427.2684	AUC	N-stearoyl arginine	LMFA08020136
443.316	VIP & RF	N-palmitoyl tryptophan	LMFA08020095
450.2742	VIP	Glycochenodeoxycholic acid	LMST05030008
522.5724	AUC	LysoPC(18:1)	LMGP01050032
576.3644	VIP & AUC	LysoPC(22:2)	LMGP01050135
620.3867	AUC	PE(P-16:0/12:0)	LMGP02030007
628.4482	VIP	Cer(t18:0/20:0(2OH))	LMSP02030017

664.4095	AUC	PE(30:0)	LMGP02010301
672.4706	VIP & AUC	PE(31:3)	LMGP02010392
679.4568	RF	PA(P-16:0/20:5)	LMGP10030021
708.4333	VIP & AUC	Cer(d18:0/28:0)	LMSP02020049
713.3851	AUC	CerPE(d14:2/24:1)	LMSP03020037
716.4931	RF	PE(24:2)	LMGP02011198
729.3583	VIP & RF & AUC	PI 23:2;O3	LMGP20050011
801.4294	RF & AUC	PI(32:5)	LMGP06010617
832.5123	VIP & RF	PS(40:8)	LMGP03010858
840.4976	VIP &RF & AUC	PE(44:10)	LMGP02011272
845.4516	RF & AUC	PI(35:4)	LMGP06010059
864.5524	VIP	PS(42:6)	LMGP03010861
869.5070	VIP	PI(O-16:0/22:6)	LMGP06020089
889.4723	VIP & RF& AUC	PG(44:1)	LMGP04010722
892.5812	VIP & AUC	PS(44:6)	LMGP03010973
897.5344	AUC	PI(39:6)	LMGP06010863
